# Blended Cement Mixed with Basic Oxygen Steelmaking Slag (BOF) as an Alternative Green Building Material

**DOI:** 10.3390/ma13143062

**Published:** 2020-07-09

**Authors:** Assel Jexembayeva, Talal Salem, Pengcheng Jiao, Bozhi Hou, Rimma Niyazbekova

**Affiliations:** 1 Department of Civil and Environmental Engineering, Michigan State University, East Lansing, MI 48824, USA; jexembay@msu.edu (A.J.); salemtal@msu.edu (T.S.); 2 Technical Faculty, Saken Seifullin Kazakh Agro Technical University, Astana 010011, Kazakhstan; rimma.n60@mail.ru; 3 Institute of Port, Coastal and Offshore Engineering, Ocean College, Zhejiang University, Zhoushan 316021, China; 3160100620@zju.edu.cn; 4 Engineering Research Center of Oceanic Sensing Technology and Equipment, Zhejiang University, Ministry of Education, Hangzhou 310000, China

**Keywords:** blended Portland slag cement, basic oxygen steelmaking slag (BOF), ordinary Portland cement (OPC), mechanical and chemical properties

## Abstract

Portland cement tends to exhibit negative environmental impacts; thus, it is required to find measures that will improve its green credentials. In this study, we report a blended Portland slag cement as an alternative environmentally-friendly building material in order to reduce the total carbon footprint resulted from the production of the ordinary Portland cement (OPC), which may resolve the environmental issues associated with carbon dioxide emissions. The ordinary Portland cement type I enhanced by basic oxygen steelmaking slag (BOF) is produced and casted into cubic and beam-like samples for the compressive and three-point bending tests, and the compressive and flexural strengths are experimentally measured. Numerical simulations are conducted to compare with the experimental result and satisfactory agreements are obtained. X-ray diffraction (XRD) investigations and porosity tests are then carried out using the semi-adiabatic calorimetry, which indicates that 5% BOF is the optimal ratio to accelerate the hydration process while increasing the amount of hydration products, especially at the early curing age of 3 days. Scanning electron microscope (SEM) images further indicate that BOF can be used to prevent the development of microcracks while mitigating their propagation within cement mortar. Our study indicates that the compressive strength of OPC can be critically increased by BOF at the relatively low concentrations of 5%. The blended slag cement reported in this paper provides advanced understanding on the green building material that uses byproduct wastes for the mechanical and electrical performance.

## 1. Introduction

Critical environmental impacts are reported from the production of Portland cement, e.g., approximately 5% of energy use and 10% of anthropogenic CO_2_ emissions are associated with production of ordinary Portland cement (OPC), and more than 30 billion tons of natural resources are consumed for cement production [1,2], especially given the fact that over 1.6 billion metric tons of cement are produced every year [3,4]. To resolve the environmental issues resulted in cement, solutions might be achieved from two perspectives: (1) increasing the functionality and sustainability of concrete-based infrastructures, and (2) fabricating cement using by-product wastes. Ideally, improving the overall performance while maximizing the application of market-limited industrial wastes lead to the emerging sustainable Portland cement in the cement and concrete industries [5,6,7,8]. Studies have been reported to partial replace Portland cement with diverse industrial byproduct raw materials, such as basic oxygen steelmaking slag (BOF) [9,10,11]. Steel production is estimated to be 50 million tons worldwide every year, which brings tons of steel byproducts [12,13,14]. China, the largest steel market worldwide that produces more than one-half of the global raw steel production in 2019, has shifted its emphasis on the effective application of steel byproducts, such as BOF, in production of Portland cement [15]. To effectively produce and apply byproducts-enabled Portland cement, it is necessary to investigate the physical, chemical, and mechanical properties of the cement.

Studies have been conducted to evaluate the physical and chemical properties of different types of BOFs [16], as well as the usage of BOF as replacement in cement and silicate concretes [17,18]. BOF has been successfully used as a construction aggregate in many cement applications, including asphaltic concrete, Portland cement concrete, roadway embankment, shoulders and on unpaved roads, road base material, walkways, and driveways [19,20,21,22]. Studies have been conducted to investigate the properties of Portland cement enhanced by BOF additions. Liu and Li [23] investigated the effect of using ground BOF on the mechanical properties of concrete and found that 20% or more BOF resulted in the decreasing of the strength for the concrete at the early ages. Lin et al. [24] demonstrated the enhancement of the compressive strength by using 5–20% municipal solid waste incineration-light emitting diode slag. To satisfy the strength requirements, the usage of BOF in cement production is mainly focused on the relatively low percentage (i.e., approximately 10%) [25]. Tsakiridis et al. [26] reported that 10.5% was the optimum replacement dosage for BOF in production of blended slag cement. Monshi and Asgarani [27] proposed a cement composition where 49% iron slag, 43% calcined lime, and 8% steel slag were used to produce the Portland cement that satisfied the mechanical properties (i.e., compressive strength). Wang et al. [28] used steel slag as a composite mineral admixture in cement-based materials and found that high content of steel slag may have some negative impacts on the mechanical properties of concrete at the late ages. High-performance asphalt concrete mixtures were reported based on high-strength BOF in the U.S. state of Colorado [29], and studies have been carried out using BOF in agricultural (non-construction) applications, such as soil demineralization [30,31]. More recently, studies have been conducted to investigate the advanced performance of blended cement [32,33,34,35,36]. Zhang et al. [37] investigated the use of the BOF by studying the effect of the slag product and iron concentrate (products resulted from the magnetic separation process performed on the BOF). The results show that, blended cements with residual slag product have comparable properties with Portland cement which may have big economic impact and successful achievements in reduction of CO_2_ emissions and energy conservation. Carvalho et al. studied the partial replacement of ground granulated blast furnace slag (GGBF) by BOF slag in the production of slag Portland cement [38] and as a partial replacement of the clinker [39]. The optimal 5.4% BOF replacement content was reported in terms of the increment in the compressive strength exceeding the standards required by the ASTM C595 [40]. Lu et al. studied the usage of BOF slag in Portland cement mortars, and the authors found that 20% of BOF will significantly reduce the compressive strength and slightly increased the volume expansion of the mortars. However, when the BOF was alkaline activated through the adding of NaOH and Na_2_SiO_3_, the mortars were close to the Portland cement mortars [31].

Here, we report blended Portland slag cement, in which ordinary Portland cement was enhanced by basic oxygen steelmaking slag (BOF). The environment-friendly, self-sensing concrete reported in Reference [41] is expanded for advanced understanding on the mechanical and chemical properties of the BOF-enhanced cement. The enhanced physical, chemical, and mechanical characteristics are investigated, and the optimal dosage of 5% BOF is obtained for the BOF-enhanced cement. The components and production of the BOF-enhanced cement are first reported, and experiments are then carried out to obtain the characteristics of the BOF-enhanced cement, including the physical performance (i.e., particle size distributions of BOF and OPC), the chemical performance (i.e., XRD and semi-adiabatic calorimetry results of OPC and BOF), and the mechanical performance (i.e., compressive and flexural strengths). Numerical simulations are conducted to validate the mechanical response of the BOF-enhanced cement, and satisfactory agreements are obtained. The blended Portland slag cement reported in this paper leads to the advanced understanding on the environment-friendly blended cements that uses byproduct wastes for the mechanical and electrical performance.

## 2. Components, Production, and Experimental Testing of BOF-Enhanced Cement

### 2.1. Components of BOF-Enhanced Cement Mortar

BOF was used to partially replace the ordinary Portland cement (OPC) type I with different dosages and experimentally calibrate the enhanced mechanical properties. Note that BOF is the waste of steel production obtained by melting cast iron with lime or dolomite flux in the gaseous oxygen medium. The impurities in cast iron are mainly the carbon, phosphorus, silicon, and manganese, which can be indicated as

(1) $$C\to {\mathrm{CO}}_{2},\;P\to P_{2}O_{5},\;\mathrm{Si}\to {\mathrm{SiO}}_{2},\;\mathrm{and}\;\mathrm{Mn}\to \mathrm{MnO},$$

where carbon dioxide is volatilized and other oxides (i.e., iron, silicon, manganese oxides) are combined with the lime or dolomite that are available in the slag. Therefore, BOF can be used as positive mixture to Portland cement. BOF has calcium oxide as its dominant compound, according to the chemical composition presented in Table 1. In particular, BOF with 1%, 3%, 5%, 10%, and 15% weight ratios of Portland cement type I are used to produce the blended cement mortar samples, and the BOF ratios are selected based on the optimum dosage of the steel slag. BOF is obtained from JSC “ArcelorMittal Temirtau” (Temirtau, Kazakhstan) “ArcelorMittal Temirtau” with the pH of 12.07, the conductivity of 1087 µs/cm, and the specific gravity of 2.09. OPC type I is obtained from Alpena cement plant in the U.S. state of Michigan, with the Blaine fineness of 372 m^2^/kg, the air content of 8% and the autoclave expansion of 0.05%. BOF is the by-product of molten iron processing, which has different types of steel slags depending on the type (grade) of steel and the furnace being used during the production process [42]. BOF used in the experiments had the aluminum oxides-to-silica oxides weight ratio of 24.37%, and the calcium oxide-to-silica oxide weight fraction of 3.2. BOF was first grinded by the Cryogenic grinding method using the liquid nitrogen machine “Micron powder system” to obtain the mean particle size of 16 μm.

### 2.2. Production of BOF-Enhanced Cement Mortar

**Figure 1:**
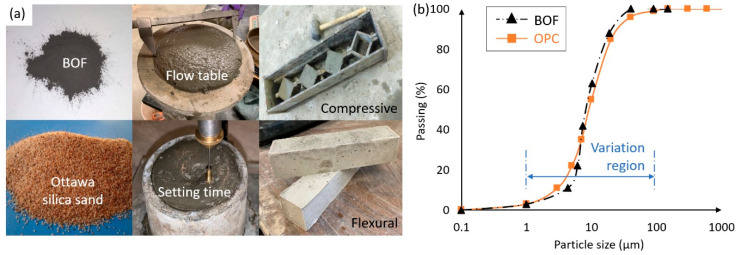
(**a**) Production of the BOF-enhanced cement mortar. (**b**) Particle size distributions of BOF and OPC in the BOF-enhanced cement.

Figure 1a presents the production processes of the BOF-enhanced cement mortar. The BOF-enhanced cement and OPC mortars were shacked on the vibrating table to reduce the air bubbles while ensuring the compaction. After 24 h, the specimens were demolded and placed into the curing room under the temperature of 20 °C and the relative humidity of 95%. The semi-adiabatic calorimetry was used to determine the hydration heat-induced temperature increasing of the BOF-enhanced cement under the room temperature of 25 °C. The water to cement *(W/C)* ratio was specified following the requirements in ASTM C305 [43] and immediately placed inside the adiabatic chamber. The temperature development of the fresh BOF-enhanced cement and OPC specimens was monitored. The initial and final setting times of the BOF-enhanced and OPC mortar samples were measured following ASTM C191 [44] using the Vicat needle apparatus [45]. The amount of water mixed with the cement was selected to produce a normal consistency per ASTM C187 [46]. To sum up, the production process of the BOF-enhanced cement mortar included three steps:

(1) Supplementary cementing materials were mixed with the OPC for 3 min at the low speed the using classic™ quart tilt-head stand mixer to ensure the pervasion of moisture over the whole particles;
(2) Water was added to the mixed OPC, and the W/C ratio was adjusted to produce a fresh mix flow of 110 ± 5% per ASTM C1437 [47];
(3) Ottawa silica sand was slowly added to the mixed materials to meet the sand-to-cement ratio of 2.75, and mixed for 30 s. The mixed cement samples were kept for 90 s and then stirred at the medium speed for 60 s.

Fine BOF were used as nanostructure replacement of cement to improve the interaction between the OPC and BOF, while increasing the hydration rate [48]. The particle size distributions of BOF and OPC in the BOF-enhanced cement was measured via the 3071A Analyzer, as shown in Figure 1b. It can be seen that significant variations of the passing ratio were obtained for the particle sizes of BOF and OPC between 1 and 100 µm.

**Figure 2:**
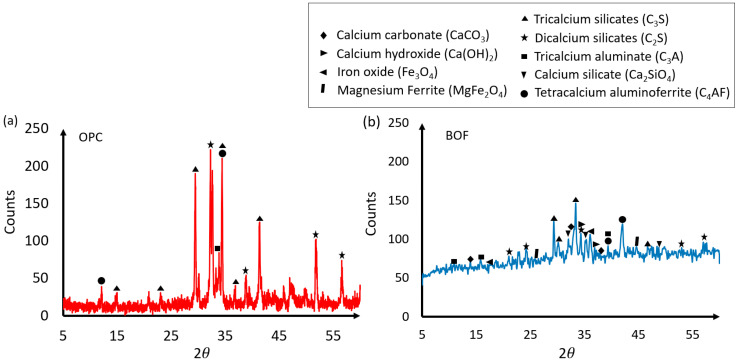
XRD results of (**a**) OPC and (**b**) BOF in the BOF-enhanced cement.

The mineralogy of the BOF and OPC under consideration were assessed using XRD technique. A Bruker D8 X-ray diffractometer (Bruker, Karlsruhe, Germany) equipped with Cu x-ray radiation operating at 40kV and 30mA was particularly used to conduct the XRD tests at the rate of 2°/min, covering a reflection angle range 2*θ* of 5−60°. The XRD results of OPC and BOF in the BOF-enhanced cement are presented in Figure 2. It can be seen that OPC mainly contains tricalcium silicates (C_3_S), dicalcium silicates (C_2_S) and tetracalcium aluminoferrite (C_4_AF), and BOF mainly has calcium carbonate (CaCO_3_), calcium hydroxide (Ca(OH)_2_), iron oxide (Fe_3_O_4_), tricalcium silicates (C_3_S), dicalcium silicates (C_2_S) and tetracalcium aluminoferrite (C_4_AF). Comparing Figure 2a,b, CaCO_3_, Ca(OH)_2_, and Fe_3_O_4_ lead to the main difference between OPC and BOF.

### 2.3. Experimental Procedures

**Figure 3:**
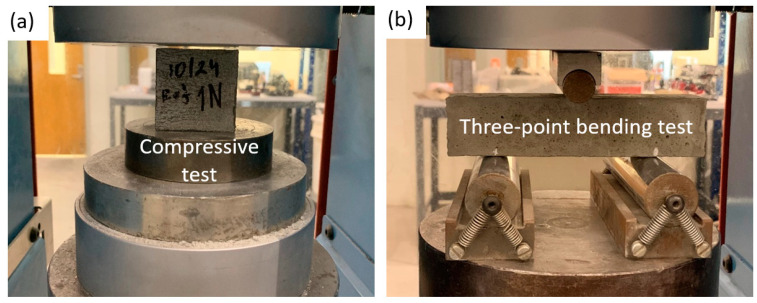
Experimental setup for the (**a**) compressive and (**b**) three-point bending tests (all scale bars are 20 mm).

In this study, the compressive and bending testing were carried out to experimentally measure the mechanical properties of the BOF-enhanced cement mortar (i.e., compressive and flexural strengths), and the OPC were tested and compared to the reference. A total of three specimens were tested for each geometry set using the FORNEY compression machine to obtain the average strength. Figure 3 presents the experimental setup for the compressive and bending tests. Figure 3a,b, respectively, demonstrate the compressive and three-point bending tests. The compressive strength was measured for 50 mm cubic BOF-enhanced cement mortar specimens under the curing time of 3, 7, and 28 days following ASTM C109 [49], and the flexural strength were obtained for 40 mm×40 mm×160 mm prism molds following ASTM C348 [50]. The microstructural characteristics of the blended cement pastes with the 1%, 3%, 5%, 10%, and 15% dosages of BOF were particularly evaluated by the X-ray diffraction (XRD) analysis, a scanning electron microscope (Saken Seifullin Kazakh Agro Technical University, Nur-Sultan, Kazakhstan), and semi-adiabatic calorimetry test (Michigan State University, East Lansing, MI, USA). The scanning electron microscope (SEM) images of the hydration products were captured using the Hitachi TM3030 (Hitachi, Tokyo, Japan), and the Bruker XFlash MIN SVE microanalysis system (Bruker Nano GmbH, Berlin, Germany) was used to assess the microstructural attributes and microcrack conditions of the BOF-enhanced cement pastes. Note that the cement specimens were imaged in high-vacuum mode at the accelerating voltage of 15 kV.

### 2.4. Mechanical Results and Discussion

**Figure 4:**
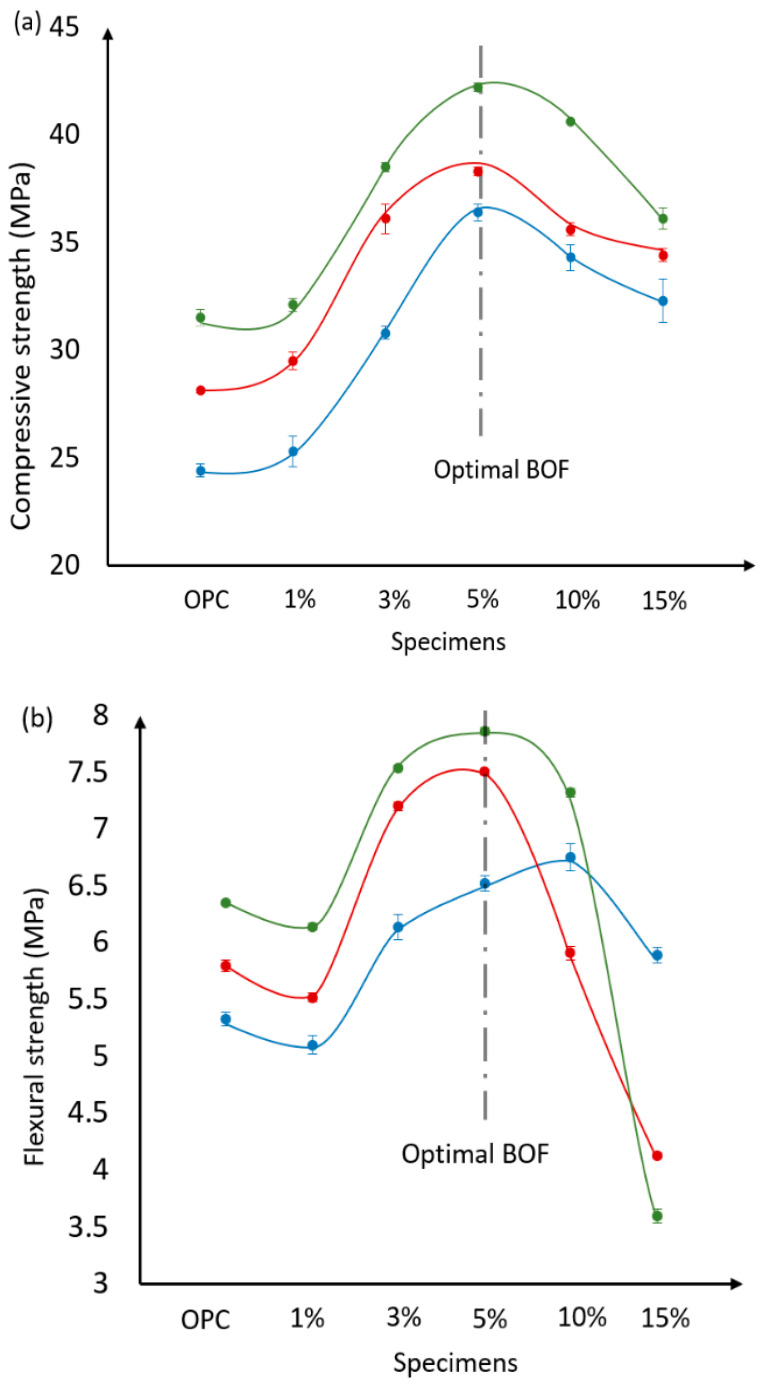
Distribution trends of the (**a**) compressive and (**b**) flexural strengths between the OPC and BOF-enabled cement.

Table 2 presents the compressive, flexural strengths and the water-to-cement ratio of the BOF-enhanced cement mortar specimens with different dosages of BOF (i.e., 1%, 3%, 5%, 10%, and 15% BOF) under the curing time of 3, 7, and 28 days. To compare of BOF-induced strength enhancement, the compressive and flexural strengths of the OPC mortar specimens are provided. It can be seen that, for example, about 50% increment of the compressive strength is obtained for the 5% BOF cement at the early age (i.e., 3 days), which is reduced to about 34% increment at the late age of 28 days. This is because the fineness of the BOF (microparticles) tends to react faster and increase the early and late compressive strengths of the BOF-enhanced cement samples by improving the microstructures of the cement stone [41]. Using 10% and 15% BOF results in the reversed trends for the early and late ages (i.e., reduction in compressive strength), which are estimated to be 3.1% and 14.4% at the age of 28 days, respectively, compared with the optimum dosage of 5% BOF. This was also reported by Shi et al. [51], in which the authors investigated the properties of the cement mortar with a superfine steel slag and found that the compressive strength of the cement mortar was continuously decreased when the replacement weight ratio of the superfine steel slag was above 10%. The flexural strength of the BOF-enhanced cement mortar with 1% BOF was reduced under the early and late curing conditions (i.e., 3, 7, and 28 days). Decreasing of the flexural strength is also obtained for 15% BOF under the curing time of 7 and 28 days. This phenomenon can be explained by the presence of the CaCO_3_, Ca(OH)_2_, and Fe_3_O_4_ in BOF, as shown in Figure 2. In addition, the relatively high content of Fe_3_O_4_ in BOF may has negative affect on the final hardening of the BOF-enhanced cement [51]. In addition, Schuldyakova et al. observed a similar trend (i.e., a reduction at the early age flexural strength) when the substitution level of cement by blast furnace slag was increased [52]. The authors explained that the phenomenon was due to the increase of the water demand as the slag dosage increase. In general, 5% BOF is observed to be the optimum dosage for the BOF-enhanced cement. Based on the experimental results in Table 2, Figure 4 demonstrates the distribution trends of the compressive and flexural strengths between the OPC and BOF-enabled cement.

**Figure 5:**
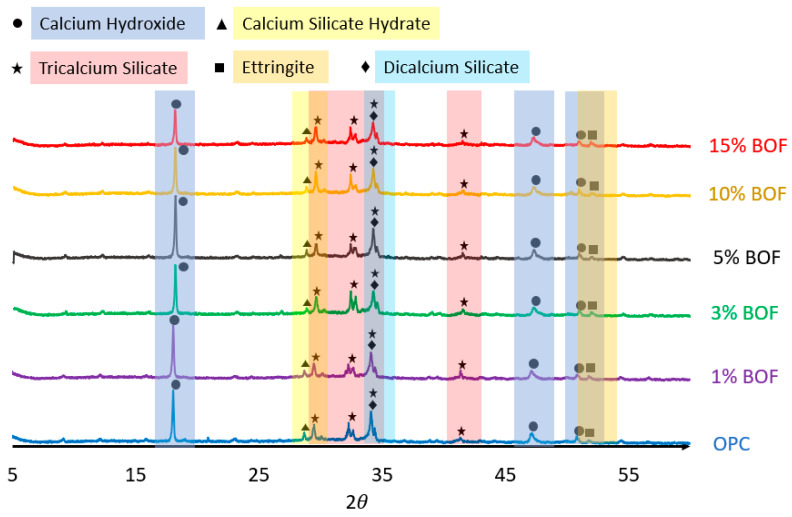
XRD results of the OPC and BOF-enhanced cement mortar with 1%, 3%, 5%, 10%, and 15% BOF at the early curing age of 3 days.

Next, the XRD analysis of the BOF-enhanced cement is obtained at the early age of 3 days, as shown in Figure 5. It can be seen that, for the BOF-enhanced cement with 1–15% BOF, the main mineralogical phases were calcium silicate hydrate (C-S-H), tricalcium silicate (C_3_S), dicalcium silicate (C_2_S), ettringite (E), and calcium hydroxide (CH), which were formed in the significant quantities at the early age [53,54]. In particular, the variation in the characteristic peak of C_3_S at 2θ=29° was less sharp in the case of 5% BOF, which explains the optimum conversion of the C_3_S-to-C-S-H gel during the hydration reaction [55]. However, other peaks are remained unchanged at 5% BOF, which results in the increasing of the compressive and flexural strengths for the BOF-enhanced cement.

**Figure 6:**
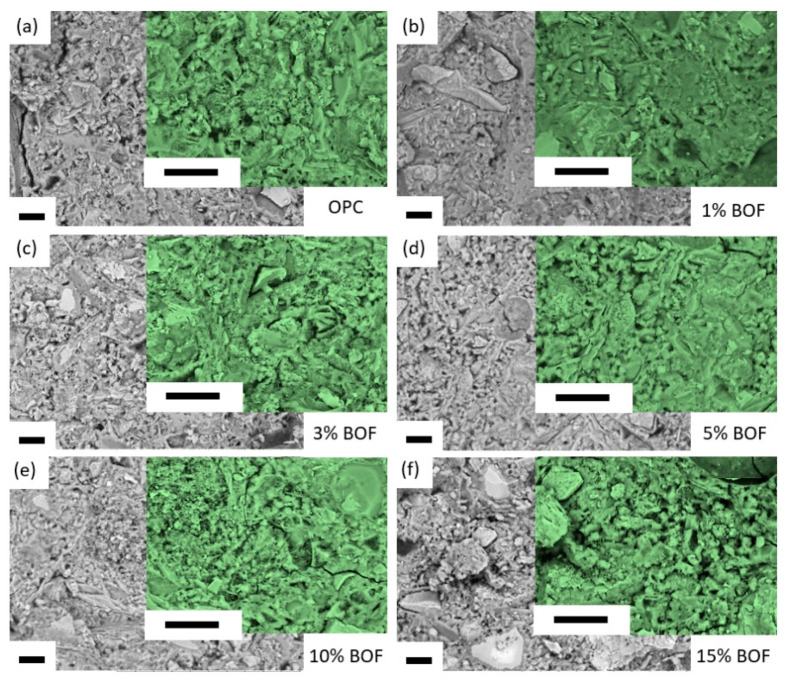
SEM images for (**a**) OPC, as well as BOF-enhanced cement mortar with (**b**) 1% BOF, (**c**) 3% BOF, (**d**) 5% BOF, (**e**) 10% BOF, and (**f**) 15% BOF, at the curing age of 28 days (gray images are the low magnification of 1000×, and green images are the high magnification of 2000×) (all scale bars are 100 µm).

Figure 6 presents the SEM images of the BOF-enhanced cement mortar specimens at the late age of 28 days with the relatively lower magnification (i.e., 1000×) and higher magnification (i.e., 2000×). In general, high magnification images are obtained with certain slag clusters. Figure 6a shows that the OPC cement has a homogeneous structure. This could be due to the calcium silicate hydrate (CSH) gel fibers form denser overlap to the network structure and connect with the surrounding unhydrated cement particles through the hexagonal CH crystals, which tends to form a framework by staggering [56]. Similar observation is obtained in Figure 6b, which can be explained by the low amount of 1% BOF in the BOF-enhanced cement. Figure 6c shows an increment of CH crystallohydrates intertwined with hydrated plates of C-S-H gel and needle-shaped ettringite. The inter-tissue spaces inside the paste frame are filled by CH crystals and C-S-H gel in 5% BOF in Figure 6d, which explains the formation of a dense crystallized structure [57]. Figure 6e,f are observed with the loose structures that have noticeable pores with less dense network structures.

**Figure 7:**
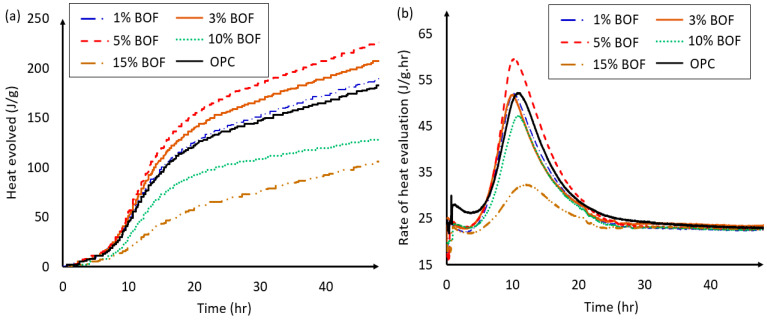
(**a**) Heat evolved and (**b**) rate of heat evaluation of the semi-adiabatic calorimetry tests for OPC and BOF-enhanced cement with 1%, 3%, 5%, 10%, and 15% BOF.

Figure 7 presents the heat evolved and rate of heat evaluation of the semi-adiabatic tests of the OPC and BOF-enhanced cement pastes with 1%, 3%, 5%, 10%, and 15% BOF. In general, the heat evolution can be divided into five periods, (i) initial reaction, (ii) induction period, (iii) acceleratory period, (iv) decelerating period, and (v) period of slow and continued reaction. A rapid heat evolution is observed during the initial reaction period, which is due to the rapid formation of an amorphous layer of hydration product around the cement particles, which separates them from the pore solution and prevents further rapid dissolution. Followed by the induction period where almost no reaction occurs, the rate of heat evolution drops to a very low value. During the third period, the rate of reaction increases rapidly, reaching a maximum (usually within 24 h). This is mainly due to the hydration of the C3S and fast formation of C–S–H gel. It is worth mentioning that the rate of hydration in this period is controlled by the rate at which the hydration products nucleate and grow. When the decelerating period takes place, a heat flow is observed, which could be due to renewed C_3_A dissolution and ettringite precipitation. Reaching to period of slow and continued reaction, also known as diffusion-limited reaction period, the diffusion processes become slower as the layer of hydration product around the cement particles becomes thicker [58,59]. Figure 7a shows that the heat hydration rates of the OPC and BOF-enhanced cement with 1% and 3% BOF are obtained as around 51.7 J/g. Five percent BOF, however, results in the highest peak with the increasing of the heat released rate by 13.3%. This increment could be explained due to the fact that the temperature increasing accelerates the hydration reactions of the BOF-enhanced cement, which leads to the production of more C-S-H gel, and, therefore, increases the strength of the cement samples at the early age. This is coincided with the findings reported in Reference [48]. BOF microparticles and their chemical composition are likely to play a significant role in enhancing the activation of the hydration process [60]. Figure 7b indicates the reduction of heat evaluation rate for BOF-enhanced cement the with 10% and 15% BOF, which can be considered as an indicator of slowing down the hydration process of the cement composition, and results in the decreasing of the compressive and flexural strengths in those cement samples (see Table 3). This finding can also be explained by the setting times presented in Table 3. In particular, increasing the dosage of the BOF increases the initial and final setting times, which is because the retardation of the hardening process caused by the lack of the cement and low hydration rate of additives [61]. In the case of 1% BOF, the initial and final setting times are observed to be similar. When 3% and 5% BOF are used, the hydration process became slower, which may be due to the hidden properties of BOF and fineness of grinding. For 5% BOF, the initial and final setting times was increased by 85% and 96%, respectively, when compared with the OPC. BOF influences the results by increasing the released Ca^+2^, which affects the growth of crystals during the final hardening of the cement [62]. Further increase in the dosage of 10% BOF, 15% will significantly slow down the hardening process, which is caused a delay in the strength gain. It is worth mentioning that an extended setting time could be an advantage to the application, which required longer workable period [63].

## 3. Numerical Simulations for the OPC

### 3.1. Numerical Modeling

In this section, the numerical models are developed in ABAQUS v6.14-1 (Dassault Systèmes Simulia., Providence, RI, USA) to obtain the mechanical response of the OPC cured after 28 days. We present the finite element (FE) models to compare with the experimental results of the compressive and flexural strengths of the OPC specimens presented in Table 2 (i.e., the values in bold and italic) in order to check the experimental setup.

**Figure 8:**
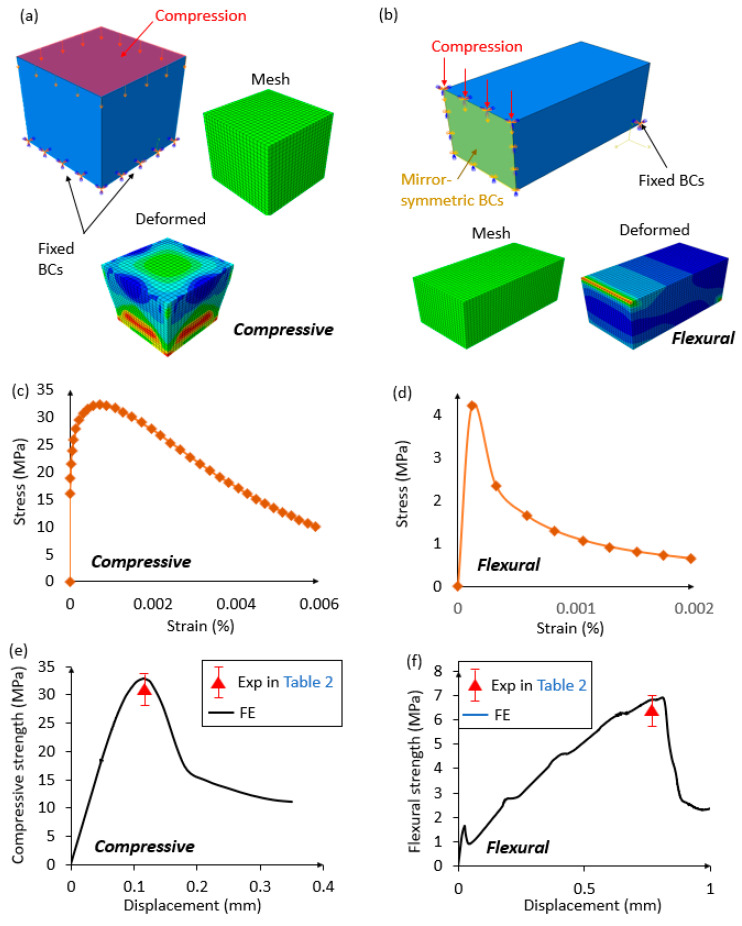
Loading and boundary conditions, mesh and deformed configurations of the OPC in the (**a**) compressive and (**b**) flexural testing. Experimentally measured (**c**) compressive and (**d**) tensile behaviors of the OPC specimens. Comparisons of the (**e**) compressive strength-displacement relation and (**f**) flexural strength-displacement relation for the OPC cured after 28 days between the experimental and numerical results (the experimental results are presented in Table 2 in bold and italic).

Figure 8a,b, respectively, show the mesh, loading, and boundary conditions of the numerical models in the compressive and three-point bending tests. The concrete damaged plasticity (CDP) model is used to define the material properties of the OPC in the FE models, and the dynamic implicit algorithm with the solid element (C3D8R) is applied. In particular, the parameters of the dilation angle, eccentricity, $\frac{f_{b0}}{f_{c0}}$, *K*, and viscosity are determined following the study [64]. Figure 8c,d present the experimentally measured compressive and tensile behaviors of the OPC, respectively, which are comparable with the results presented in the existing study [65]. The compressive and tensile relations are used to define the OPC in the FE models. Due to the symmetry of the three-point bending testing, only half of the cement samples are considered. Displacement-control loading conditions are used for the compressive and flexural tests. The geometric and material properties, as well as the mesh and loading conditions, of the FE models are listed in Table 4.

### 3.2. Comparison between the Experimental and Numerical Results and Disucssion

Figure 8e,f compare the compressive and flexural strengths, respectively, between the experimental and numerical results for the OPC cured after 28 days. The experimental results of the compressive and flexural strengths are 31.5 ± 0.4 MPa, and 6.35 ± 0.02 MPa, respectively (i.e., the bold and italic values in Table 2). It can be seen that the compressive and flexural strengths are obtained with good agreements between the experimental and numerical results.

**Figure 9:**
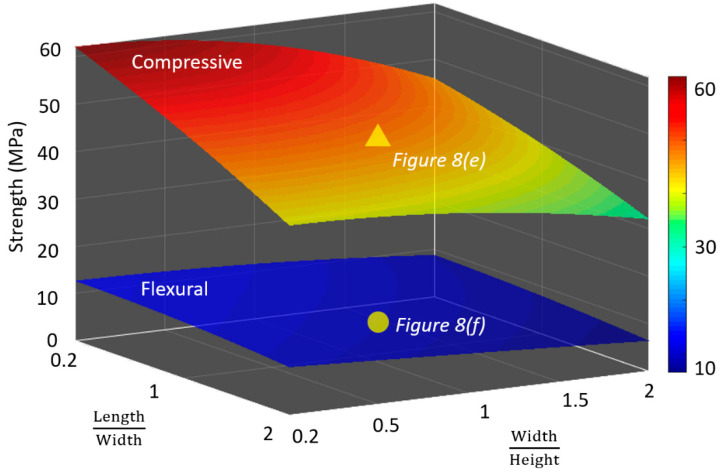
Variations of the compressive and flexural strengths of the OPC with respect to the length-to-width and width-to-height ratios.

Considering the expensive and time-consuming characteristics of the experiments for the OPC, it is typically more efficient to numerically calculate and predict the compressive and flexural strengths of the OPC. Parametric studies are conducted using the numerical model to investigate the influences of the geometric parameter ratios (i.e., length-to-width and width-to-height ratios) on the compressive and flexural strengths of the OPC. Figure 9 presents the distributions of the compressive and flexural strengths with respect to the length-to-width and width-to-height ratios. It can be seen that the compressive and flexural strengths are affected by the ratios. However, the variation of the compressive strength is more significant than that of the flexural strength.

## 4. Discussion and Future Work

This study presents the BOF enhanced Portland cement that can be used as an alternative green building material. In particular, the compressive and flexural strengths and the heat-related properties of the BOF-enhanced cement are investigated to expand the advanced understanding on the environment-friendly, self-sensing concrete [41]. The proposed cement embodies the abundant calcium silicates presented in the BOF slag to yield a high-performance green blended cement for concrete production and other applications. This blended slag cement offers significant advantages over OPC in terms of:

- Carbon footprint and cost: Replacing Portland cement with BOF helps in lowering the total carbon footprint associated with the production of the OPC (about 800 kg per ton) [66]. A recent statistical study in 2019 showed that one ton of OPC cost about US$ 123 [67]; therefore, replacing OPC with industrial waste material (BOF) will result in reducing the total cost.
- Mechanical performance and stiffening: The conducted experiments indicated that an optimum dosage of 5% BOF will result in better mechanical performance in terms of compressive and flexural strengths. Adding BOF will extend both initial and final setting times, which opens doors for new applications that require longer time of setting for the concrete.

However, it is worthwhile to point out that further efforts are needed to develop the numerical models on the BOF-enhanced cement. Future work is needed in Figure 6 to obtain the BSM images to investigate the microstructures of the BOF-enhanced cement. Ca/Si ratio relations are needed to consider with respect to the strengths of the BOF-enhanced cement [32]. In addition, the study can be continued by extending the series of tests on the new cement obtained by adding BOF, as well as continuing the research by tests, such as: determination of setting time, determination of stability, etc. Analysis with modern designs and optimization methods for the fabrication and surface methodology in the experiments are necessary in the future, which is capable of capturing the effects of influential parameters on the mechanical and chemical properties of the reported BOF-enhanced cement.

## 5. Conclusions

An investigation was conducted in order to assess the effects of adding BOF slag in 1, 3, 5, 10, and 15% for periods up to 28 days on the mechanical and chemical characteristics of the BOF-blended cement. The effects of adding BOF on the cement strength development characteristics, microstructure, crystallinity, hydration behavior, and setting time of the resulting blended cement pastes were experimentally evaluated. In addition, numerical models were developed to compare with the experimental results, and satisfactory agreements were obtained. The following primary conclusions were derived based on the data obtained in this study.

- Adding a relatively small amount of BOF (< 15% of cement weight) had positive effects in terms of compressive strength for both early and late ages; however, 5% BOF was found to be the optimal dosage to accelerate the strength development. The influence of the BOF on the flexural strength showed different trend, in which 10 and 5% of BOF was reported to have highest effects on strength development at early and late ages, respectively.
- The crystallinity of the Portland cement did not experience notable changes when a relatively small amount of BOF was added. Some consumption of the tricalcium silicates was observed, which could due to the conversion of the tricalcium silicates to calcium silicate hydrates. Enhanced cement pastes with 3% and 5% BOF experienced less microcracking when subjected to vacuum drying comparing with the ordinary Portland cement.
- Introducing 5% of BOF positively affected the hydration kinetics of the Portland cement pastes. However, initial and final setting times were observed to be longer when BOF was used.

## Figures and Tables

**Table 1:** Chemical composition and loss on the ignition results (wt.%) of the basic oxygen steelmaking slag (BOF) and ordinary Portland cement (OPC).

Contents	SiO_2_	CaO	Al_2_O_3_	Fe_2_O_3_	MgO	MnO	SO_3_	TiO_2_	P_2_O_5_	LOI
OPC	19.94	64.20	4.86	3.15	2.71	2.83	1.67	–	–	2.5
BOF	12.03	46.17	1.53	21.66	4.53	5.10	0.77	0.58	2.52	2.3

**Table 2:** Compressive and flexural strengths for BOF-enhanced cement and OPC mortar specimens with associated standard error (standard deviation).

Specimens	W/C	Compressive (MPa)			Flexural (MPa)		
		3 days	7 days	28 days	3 days	7 days	28 days
**Ordinary Portland Cement (OPC)**	0.26	24.4 ± 0.3	28.1 ± 0.1	31.5 ± 0.4 (Figure 8e)	5.33 ± 0.06	5.80 ± 0.05	6.35 ± 0.02 (Figure 8f)
1% BOF	0.26	25.3 ± 0.7	29.5 ± 0.4	32.1 ± 0.3	5.10 ± 0.08	5.52 ± 0.04	6.14 ± 0.03
3% BOF	0.26	30.8 ± 0.3	36.1 ± 0.7	38.5 ± 0.2	6.14 ± 0.11	7.20 ± 0.04	7.54 ± 0.02
***5%*** BOF	0.27	36.4 ± 0.4	38.3 ± 0.2	42.2 ± 0.2	6.52 ± 0.07	7.50 ± 0.02	7.86 ± 0.03
10% BOF	0.28	34.3 ± 0.6	35.6 ± 0.3	40.6 ± 0.1	6.75 ± 0.12	5.91 ± 0.06	7.32 ± 0.04
15% BOF	0.28	32.3 ± 0.3	34.4 ± 0.3	36.1 ± 0.5	5.89 ± 0.07	4.13 ± 0.02	3.60 ± 0.06

**Table 3:** Initial and final setting times for OPC and BOF-enhanced cement mortars (all in min).

Specimens	Initial	Final
Ordinary Portland cement (OPC)	150	255
BOF-enhanced cement (1% BOF)	152	257
3% BOF	212	380
5% BOF	277	500
10% BOF	342	620
15% BOF	407	741

**Table 4:** Geometric and material properties, as well as the mesh and loading conditions, for the OPC specimens.

**Geometric Property**	Compressive (mm)	Length *L*	40
		Width *b*	40
		Height *h*	40
	Flexural (mm)	Length *L*	160
		Width *b*	40
		Height *h*	40
**Material Property**	Density	*ρ* (kg/m^3^)	2300
	Young’s modulus Poisson’s ratio	*E* (GPa)	18.889
		*v*	0.18
	Concrete damaged plasticity (CDP) model [64]	Dilation angle (°)	35
		Eccentricity	0.1
		$\frac{f_{b0}}{f_{c0}}$	1.16
		K	0.667
		Viscosity parameter	0.007985
**Mesh**	Compressive Flexural	l	2
**Loading**	Compressive	Type	Displacement-control
		Displacement (mm)	0.4
		Loading time (s)	50
	Flexural	Type	Displacement-control
		Displacement (mm)	1.0
		Loading time (s)	50
